# Motor neuron disease and frontotemporal dementia: One, two, or three diseases?

**DOI:** 10.4103/0972-2327.74250

**Published:** 2010-12

**Authors:** Thomas H. Bak

**Affiliations:** Human Cognitive Neuroscience and Centre for Clinical Brain Sciences, University of Edinburgh, Edinburgh, UK

**Keywords:** Amyotrophic lateral sclerosis, cognition, frontotemporal dementia, language, motor neuron disease

## Abstract

The relationship between motor neurone disease (MND) and frontotemporal dementia (FTD) has been a topic of scientific exploration for over hundred years. A connection between both diseases was first postulated in 1932 and has been strengthened by a steady stream of case reports since then. By the late 20th century, the link between both diseases was firmly established, with the resulting condition often referred to as MND/FTD. Several strands of evidence support the notion of an MND/FTD overlap. First, a small but well-documented group of patients present with a full-blown FTD, associated with MND. Second, subtle but characteristic changes in frontal-executive functions and social cognition have been described in non-demented MND patients, often in association with frontal atrophy/hypoactivity on neuroimaging. Third, amyotrophic features have been documented in patients primarily diagnosed with FTD. Moreover, the same genetic defect can lead to FTD and MND phenotypes in different members of the same family. However, as the current research is moving toward a more fine-grained evaluation, an increasingly complex picture begins to emerge. Some features, such as psychotic symptoms or severe language deficits (particularly in comprehension and verb processing), seem to occur more often in MND/dementia than in the classical FTD. On the basis of the review of 100 years of literature as well as 10 years of clinical experience of longitudinal follow-up of MND/dementia patients, this review argues in favor of MND/dementia (or, more precisely, MND/dementia/aphasia) as a separate clinical entity, not sufficiently explained by a combination of MND and FTD.

## Introduction

The study of cognitive aspects of motor neuron disease (MND) is at the same time a very old and a very young discipline. It is old, since, as this review will demonstrate, an extensive literature on cognition and behavior in MND, dating back to the first-half of the 20th century, displays insights remarkably close to the present scientific consensus. It is young, since MND, in particular when associated with frontotemporal dementia (FTD), is at the forefront of the current radical re-classification of neurodegenerative diseases, on the basis of the latest insights from genetics, pathology, and molecular biology. The main purpose of this review is, therefore, to bring the old and the new together, to track the underlying theoretical assumptions and to explain, rather than just describe, the most influential developments. It is an ambitious task, but it is built on the hope that by understanding the past we might indeed be better equipped to face the future challenges.

## MND and FTD: A Note on the Terminology

MND and FTD share a similar terminological problem. MND is a general term for a whole group of diseases, encompassing, apart from the most common form of amyotrophic lateral sclerosis (ALS), rarer conditions such as primary lateral sclerosis (PLS) as well as progressive muscular atrophies (PMA). However, some authors, particularly in Britain, tend to use the term MND as a default, even if the clinical populations they are referring to consist entirely of ALS patients.

Similarly FTD refers to a whole group of diseases. The most common form is the so-called “frontal” or “behavioral” variant FTD (bvFTD), the less common presentations are progressive aphasias, such as nonfluent progressive aphasia (NFPA) and semantic dementia (SD). Depending on the author and the context, the term FTD might denote both the behavioral variant and the group as a whole.

In the context of this review, the majority of cases of MND/FTD can be understood as ALS/bvFTD. However, there are cases in which the distinction between different subtypes of MND and FTD plays an important role and in such situations the precise terms will be used explicitly.

## Cognitive and Behavioral Changes in MND: An Exception or a Rule?

Reports of cases combining the clinical picture of MND with mental symptoms, personality change, or dementia are almost as old as the study of MND itself. Marie[1] believed that “psychic disturbances” were fairly common in ALS. Raymond and Cestan[2] described half of their 18 ALS patients as “mentally feeble”. van Bogaert[3] in his later career, founder and first president of the World Federation of Neurology, reported in one of his early scientific works the presence of psychic alterations in 13 of 31 MND patients. Group studies were complemented by single case descriptions of patients in whom MND was associated with overt dementia.[4–7] The emerging clinical picture was clearly differentiated from the dementia of Alzheimer’s disease.[5,6] An explicit link between MND and Pick’s disease (defined clinically rather than pathologically, and therefore corresponding to the current concept of FTD), first suggested by the Munich psychiatrist Braumühl,[6] has been reproduced with remarkable regularity in subsequent studies.[8–12]

Indeed, many symptoms described in early 20th century MND patients, such as an insidious onset and slow progression of apathy, mental rigidity, greed, and an obsessive tendency to hoard things, all of it in stark contrast to the premorbid personality,[5,13] would fit well into present diagnostic criteria for FTD.[14] However, the early reports depict a wider range of psychiatric symptoms than that one would expect in typical FTD. This includes pronounced emotional changes such as depression[15] and manic-depressive illness[4] as well psychotic features such as delusions and hallucinations.[6,13] Some authors refer to the resulting clinical picture as paranoid[16] or catatonic[5] schizophrenia.

In addition to a steady flow of the further case descriptions of MND patients with psychiatric and cognitive disturbances over the following decades[8–11,17–24], two regional variants of MND/dementia became focus of particular interest from 1940s onward. One was a peculiar combination of MND, dementia, and parkinsonism, described on the Pacific island of Guam.[9,25–28] Its predominantly amyotrophic version is referred to by the indigenous Chamorro population as “Litigo”, the more parkinsonian version as “Bodig”, the mostly amnestic-demented form as “Hamalefe”. In Japan, a variant of MND associated with early and prominent dementia has been reported from the Kii Peninsula.[29–31] Interestingly, the predominance of neurofibrillary tangles on pathological examination brings both conditions together, differentiating them from the classical MND.[32]

Overall, however, apart from the two highly atypical geographical foci, the rich and detailed MND literature points to a remarkably clear and consistent picture. On one hand, cognitive and behavioral changes are not an exception: in addition to a small but well-defined subgroup of MND patients, who suffer from an overt dementia, a substantial minority of 30–50% exhibits more subtle cognitive and behavioral changes, a percentage strikingly constant from the first publications on this topic[2,3] to the latest estimates. Moreover, there is a long-standing consensus that the encountered symptoms are not random, but form a coherent clinical picture, resembling Pick’s rather than Alzheimer’s disease.[6,12] On the other hand, cognitive impairment and dementia do not form a constant feature of MND and are observed only in a subgroup of patients.

## The Cycle of Discovery and Re-discovery

The overwhelming majority of the papers discussed so far (particularly those from the first-half of the 20th century) came either from Continental Europe or from countries strongly influenced by the Continental tradition, such as Japan. Most of these papers have been published in languages other than English and remained virtually unknown in the English-speaking world, where the idea of MND as a purely motor disease sparing all higher mental functions persisted until late 20th century.

In 1981, a Canadian neurologist Arthur Hudson came across several MND patients with prominent dementia and set out to examine systematically the literature for similar cases. Thanks to the assistance of German and Japanese speaking residents at his hospital (Hudson, personal communication), he succeeded in incorporating hitherto unknown texts and making them available to the English-speaking readership. The ensuing review[33] constituted a milestone in the description of MND/dementia. Hudson documented 30 cases of MND associated with dementia and further nine cases of an association between MND, dementia and parkinsonism. The paper generated a considerable interest in this field and a little over a decade later Kew and Leigh[34] estimated the number of recorded cases of MND/dementia to be around 200.

However, written at a time when dementia was widely understood as a unitary disorder, Hudson’s paper focuses on the presence of dementia and does not attempt to characterize the nature of cognitive deficits or to differentiate between specific dementing syndromes. Indeed, for many decades dementia was virtually equalled with Alzheimer’s disease and defined mainly by a memory impairment which formed an essential part of most diagnostic criteria for dementia. Since memory deficits are not a consistent finding in MND, an amnesia-based definition will naturally lead to low estimates of dementia prevalence in this group.

The most radical shift in our understanding of dementia in MND came, therefore, through the rediscovery of Pick’s disease, now referred to as FTD, in the last decades of the 20th century. The diagnostic criteria for FTD[14] focus on behavioral changes rather than memory impairment and are, therefore, more likely to capture the nature of mental symptoms in MND. Consequently, the same Manchester group, which played a crucial role in the formulation of diagnostic criteria for FTD, revived the interest in MND/dementia and introduced the notion of MND/FTD as a nosological entity.[35] A similar tendency could be observed in United States, where one of the leading research groups on FTD directed recently their attention toward the patients with MND.[36] In a parallel line of investigation, research groups with a stronger focus on aphasia and language disorders, in Cambridge[37] and Philadelphia,[38] explored in more detail linguistic aspects of the disease. Finally, the work of Andrew Kertesz integrated different aspects of MND and FTD into the broader concept of “Pick’s Complex”.[39]

The current picture of research on MND/dementia transcends narrow disciplinary boundaries. Research groups coming from basic science lead the efforts to diagnose dementia in MND patients. Researchers originally interested in frontal-executive functions make significant contributions to our understanding of the language in MND.[40] Meetings, such as the biennial ALS/FTD meeting in London/Ontario, unite basic scientists and clinicians with a broad range of interests and expertise, including cognition, language, and behavior and lead to formulation of consensus criteria for the diagnosis of cognitive and behavioral syndromes in ALS. The field of MND/FTD research became indeed a prime example of the modern interdisciplinary approach to neuroscience.

## MND/Dementia: Clinical Picture and Natural History

The clinical descriptions of cases with MND/dementia show a remarkable consistency, both across centuries and across continents.[3,5,12,31,33,35,41–44] Indeed, in the 10 years of longitudinal follow-up of patients with various forms of neurodegeneration in the Clinic for the Disorders of Movement and Cognition in Cambridge (1996–2006), MND/dementia appeared to be the most homogeneous clinical entity, not only in terms of its symptomatology and natural course, but also in the predictability of its pathology. The following description is based, therefore, on personal clinical experience as well as on the literature.

Typically, the disease begins insidiously with changes in behavior (apathy, stereotypies), cognition (deficits in planning, forgetfulness), and/or language (dysarthria, anarthria). It is in this early stage that psychiatric symptoms, such as delusions or hallucinations are particularly frequent and prominent.[37] As the severity of the cognitive dysfunction increases, reaching the level of an overt dementia, the psychiatric symptoms seem to subside and often resolve spontaneously without medication. This could be the reason why psychotic features occur less frequently in the present than in the past descriptions of MND. The continental neurologists of early 20th century would have been, particularly in the German-speaking countries, psychiatrists at the same time as neurologists. Consequently, they are likely to have followed the patients through all the stages of their illness, from early psychiatric to late amyotrophic manifestations. In contrast, in present-day specialist clinics, the neurologists might examine the patient for the first-time months if not years after the disappearance of the florid psychotic symptoms and only a careful history taken from family members might unearth the history of delusions or hallucinations.

While the psychiatric symptoms tend to decrease, the cognitive dysfunction is becoming more and more prominent, reaching the level of overt dementia and interfering with most activities of daily life. Neuropsychological assessment reveals deficits of frontal-executive functions such as reduced verbal fluency or impaired Wisconsin Card and Weigl Block Sorting Tests, etc.[31,35] In contrast, the visuo-spatial abilities tend to be relatively well preserved.[31,35,45]

The psychiatric and cognitive stages are usually followed, within a year or two, by the classical features of MND including wasting, weakness, and fasciculations. The motor picture is usually dominated by bulbar symptoms (dysarthria, dysphagia, wasting, and fasciculations of the tongue). Wasting and weakness in the lower limbs are less frequent and many patients remain mobile until the terminal stages of the disease. The diagnosis is usually confirmed by the typical EMG pattern, not distinguishable from the classical MND. The illness progresses rapidly within 2–3 years, with aspiration pneumonia being the most common cause of death.

This characteristic sequence of symptoms (behavioral, cognitive, and amyotrophic) observed in all patients followed up in Cambridge has been described already early in this century.[5] Indeed, Brion *et al*. noticed in their extensive review of all previously published reports[12] that in the overwhelming majority of cases the mental symptoms precede the motor ones, in a minority they occur simultaneously and only rarely does the disease start with the classical MND signs. The reason for this characteristic time course remains unclear. It is not sufficiently explained by the slower progression of FTD as compared to MND, since in the MND/dementia cases the progression of dementia is much faster than in pure FTD cases.

## MND/Dementia: Pathology and Genetics

One of the most striking characteristics of MND/Dementia is the pathological homogeneity of the syndrome. All pathologically examined cases of a clinical MND/dementia that came to my knowledge during the last 15 years in Cambridge were characterized by the same combination of the classical pathological features of MND (such as neuronal loss in the anterior horn of the spine and bulbar nuclei, particularly the hypoglossal nucleus) with frontotemporal atrophy and ubiquitin-positive, tau-negative intraneuronal inclusions in the dentate fascia of the hippocampus. By contrast, and in accordance with the previous literature,[34,35,43] we observed no changes suggestive of Alzheimer’s or Lewy Body Disease.

Through the identification of ubiquitin as a crucial pathological factor in FTD and MND, the two diseases became linked at the biological as well as clinical level. Ubiquitin inclusions are meanwhile recognized as the second major pathology (apart from tauopathy) in the FTD spectrum and many cases previously referred to as “dementia lacking distinctive histology” have now been re-classified as ubiquinopathy. Interestingly, ubiquitin seems to be the most frequent pathology not only in MND/FTD, but also in semantic dementia (SD), although apparently less so in NFPA. A recent influential classification of ubiquinopathies groups patients with FTD/MND into the type 3 of ubiquitin histology, as opposed to FTD and NFPA in type 1 and SD in type 2.[47]

The next step in a fundamental re-classification of neuro-degenerative on the basis of their pathology came with the discovery of a protein called TDP-43, which has been implicated in ubiquitin inclusions. Ubiquinopathies, now redefined as “TDP-43 proteinopathies,” have been postulated as “a novel class of neurodegenerative disorders akin to α-synucleinopathies and tauopathies”.[48] Whether this classification will have a lasting effect remains to be seen—TDP-43 has been described in a wide range of disease including Alzheimer’s disease, while at the same time its genetic role in FTD and ALS has been called into question.[49] So far, no correlations have been established, neither in familial nor sporadic cases, between specific types of pathology (or gene mutations) and specific cognitive and behavioral profiles in MND patients.

Although most cases of MND/dementia are sporadic, familiar cases have been documented for a long time.[23,33,45,50–52] In his seminal review, Hudson[33] estimated that 15% of all pedigrees contained a combination of MND and dementia. Gunnarson *et al*.[52] observed a puzzling intergenerational change of phenotype in a family with an adult-onset autosomal-dominant neurodegenerative disorder. In the first three generations, the predominant phenotype was that of FTD. In the fourth generation, in contrast, only one family member developed pure dementia, four other MND. Since then, several other families with an independent occurrence of FTD and MND phenotypes within the same kindred have been described and the underlying genetic abnormality has been traced to a locus on the chromosome 9.[53–55]

## Cognitive Functions in Non-demented MND Patients

The clinical picture of MND/dementia described in the preceding section is characterized by pronounced, often dramatic symptoms. In the vast majority of MND patients, however, cognitive symptoms, if present at all, are less conspicuous, less noticed by the family and medical practitioners and often hidden behind the more obvious and incapacitating motor dysfunction. They are often unearthed only by sophisticated testing using specific cognitive tasks. However, the important issue is not so much the quantity of the symptoms as their quality. The most striking feature of the research on nondemented FTD patients is that the symptoms discovered in this group can be interpreted as the milder form of those seen in FTD/dementia.

A large study of hospital records of 255 MND patients revealed “psychic alterations” in 39 (15%), “overt dementia” in 5 (2%) of cases.[56] Subsequent studies lead to contradicting results: some described slight but significant impairment,[57,58] others found no significant difference to controls.[59,60] The picture became clearer, however, once the majority of studies focused on frontal-dysexecutive symptoms.

The cognitive test most consistently impaired in nondemented MND patients is verbal fluency.[34,36,57,61–64] Abrahams *et al*.[65] developed a special procedure to assess written verbal fluency in bulbar patients who are not able to speak. They compared the number of items produced to the time needed to write them down and concluded that the deficits cannot be attributed to a general decrease in motor speed. Other tests of frontal functions, such as Wisconsin Card Sorting Test (WCST), have also been reported as impaired in MND patients,[36,41] although the results are less consistent than those in the case of verbal fluency.

If the subtle cognitive changes observed in nondemented MND patients are interpreted as a milder form of FTD, one would also expect to find mild behavioral abnormalities as a less pronounced form of the same condition. Indeed, in the study of Lomen-Hearth *et al*.[36] all MND patients with abnormal results in verbal fluency, but also a quarter of the patients with normal word generation, displayed personality changes suggestive of FTD. This observation, suggesting that the cognitive and behavioral impairment in MND might dissociate, leads to the proposal of two distinct, albeit potentially overlapping subgroups: ALS with cognitive impairment (ALSci) and ALS with behavioral impairment (ALSbi).[36] This suggestion has been integrated into the current consensus criteria, which distinguish between a behavioral syndrome (manifesting itself through altered social conduct, emotional blunting, loss of insight, etc.) and cognitive impairment, as measured by a performance below the fifth percentile on at least two distinct cognitive tests sensitive to executive functioning. On the other hand, however, a correlation has been found between apathy, as measured on the Frontal Systems Behavior Scale (FrSBe) and verbal fluency,[38] suggesting a link between cognition and behavior.

A more recent line of evidence providing a possible link between cognitive and behavioral abnormalities is the study of emotional processing and social cognition. MND patients have been shown to be impaired on recognition of facial emotions as well as on “theory of mind” tests.[66] Moreover, comparing ALS patients to healthy as well as tetraplegic non-ALS controls, Lulé *et al*.[67] found reduced brain responses to emotional stimuli in the anterior insula, documenting a possible neural correlate for behaviorally diagnosed deficits in perception of emotions.

The clinical evidence of frontal dysfunction in MND patients is further supported by neuroimaging. Not only does neuroimaging confirm frontal atrophy[68] or frontal hypoactivation in the resting state.[69] The observed hypoperfusion or hypometabolism can be directly related to the deficit in cognitive performance. In his pioneering study, Ludolph *et al*,[63] observed a decrease in cerebral glucose metabolism in the frontal cortex of MND patients, which correlated with the performance on neuropsychological tests, particularly verbal fluency. Although the study was originally greeted with great scepticism (Ludolph, personal communication), other authors soon reported similar findings.[34] Talbot *et al*.[70] compared patients with FTD, classical MND, and MND/dementia complex and found that neuropsychological assessment and cerebral perfusion SPECT differed in severity rather than in pattern, underlying the common character of the disturbance an all three groups.

Most of the studies reported so far focused on patients with ALS–the most common and the best documented form of MND. However, in the case of primary later sclerosis (PML) the current state of evidence seems to resemble that in ALS. While the claim of “preserved higher cognitive function” has also been made in this condition, making it even into the diagnostic criteria,[71] more detailed studies detected evidence of cognitive impairment, predominantly of frontal type, in some patients.[36,72,73] Furthermore, an association of PML with full-blown (frontotemporal) dementia has also been reported.[47,74] If some authors claim that PLS cannot be clearly distinguished from ALS on the ground of its motor function and pathological findings,[75] the same can be said about its cognitive profile. With regards to other neuromuscular disorders, cognitive dysfunction has been found in different diseases with childhood onset (such as Duchenne muscular dystrophy), but not in adult-onset spinal muscular atrophy.[76]

## Language in MND: Dysarthria, Dementia, or Aphasia?

In comparison to psychiatric and frontal-executive aspects of MND, language dysfunction has received (and continues to receive) less attention. The most frequently mentioned change is a progressive reduction in spontaneous speech, often resulting in complete mutism, referred to as “speechlessness”.[3] It is tempting to attribute this phenomenon to a combination of motor (dysarthria) and psychiatric (withdrawal, apathy) factors and indeed, this has been the predominant interpretation until language in MND became focus of more systematic research in the late 20th century.[77]

However, several observations speak against such an interpretation. First, the reduction in speech production is often observed long before the emergence of the first signs of dysarthria. Second, in some patients, a period of weeks to months have been reported, in which they are not able to speak any more but can still communicate through writing. The written language in such patients is well-formed in terms of the shape of letters but severely aphasic in its content.[37] Interestingly, a documentation of orthographic errors in the Japanese kana script belongs to the earliest reports of cognitive symptoms in MND and further instances of spelling errors have been reported both from Japan[78–80] as well as Europe.[81] Spelling abnormalities can occur in patients without dementia[80] or aphasia.[79] Interestingly, dysgraphia has also been described in a study of 16 patients with PML.[73] Other abnormalities in speech production frequently observed in MND include perseverations, echolalia, and the use of stereotypic expressions.[5,50]

While language production deficits were often attributed to dysarthria, impaired language comprehension tended to be attributed to a general “loss of mind,”[13] or, in more recent terminology, to deficits in abstract reasoning.[35,82] However, more specific deficits in language comprehension do occur in some reports.[30,45] Mitsuyama[43] noticed a “severe dysphasia with poor comprehension” in one of his patients. Caselli *et al*.[77] described seven patients with a progressive nonfluent aphasia (including “significantly impaired” comprehension) as their presenting and dominant feature. Also, the five patients described by Doran *et al*.[83] showed significant deficits in syntactic comprehension, documented on the shortened version of the Token Test[84] and Test of the Reception of Grammar/TROG.[85] Furthermore, Rakowicz and Hodges[86] found an impaired TROG result in 5 out of 18 examined MND patients.

In 2001, Bak and Hodges[37] reported six MND/dementia patients with a selective deficit in the production and comprehension of verbs, as opposed to nouns and adjectives. Few years later their report has been extended to encompass six patients, four of them with the pathological confirmation of the diagnosis of MND.[37] Interestingly, the verb disadvantage was associated with prominent pathological changes in Brodmann Areas 44 and 45 (Broca’s area). Longitudinal follow-up studies confirmed the consistent nature of the selective verb impairment on consecutive testing.[87]

Moreover, tests using verbal as well as pictorial material demonstrated that the deficit is not limited to the word-level, but extends to nonverbal tasks requiring association between different actions, as opposed to the objects.[87] A recent study by Grossman *et al*.[88] confirmed a systematic deficit in action knowledge in nondemented MND patients. Although selective verb deficits have also been reported in other neurodegenerative conditions, above all in FTD,[89] recent evidence suggests that they are most pronounced in MND, constituting one of the pathognomonic cognitive features of the disease. Since the main linguistic deficits in MND patients lie in sentence comprehension and in the production and comprehension of action words, the usual brief language assessment, consisting mostly of naming of pictures of objects is the least likely tool to detect deficits in this group.

## One, Two, or Three Diseases?

In my previous reviews on cognitive aspects of MND,[37,87] the author was arguing in favor of MND and FTD as two extremes of the same continuum, a view rooted in Kertesz’s idea of “Pick’s complex” and now adopted by the majority of researchers.[36] Indeed, the concept of MND/FTD offers the most parsimonious explanation for the co-occurrence of the two conditions. It stresses not only similar clinical symptoms, but also shared molecular and genetic mechanisms, which came to light in the past decades. The already mentioned biennial meetings in London/Ontario devoted entirely to ALS/FTD overlap and attended by the leading researchers from all over the world bear witness to the popularity of this idea.

A complementary line of evidence in support of MND/FTD continuum is the observation of motor abnormalities in frontal dementias. On the pathological level, von Bagh[17] observed that 21 out of 30 cases of Pick’s disease show atrophy of the primary motor cortex and one an overt ALS. According to Constantinidis,[50] 350 cases of Pick’s disease reported in the literature 13 were diagnosed as having a combination of Pick’s disease and ALS. In a clinico-pathological study, Gustafson *et al*,[90] found fasciculations in two and autopsy evidence of MND in one of 20 patients with frontal lobe degeneration. Thus, the overlap between FTD and MND has not only been documented from the observation of frontal features in MND patients, but also amyotrophic changes in FTD patients.

The idea of MND/FTD as a single nosological entity is based on two central assumptions. First, it is assumed that the cognitive and behavioral changes seen in nondemented MND patients are a subtle form of the same, albeit more pronounced changes that characterize MND/dementia. Second, these changes must be similar in nature to those observed in classical forms of FTD. Both assumptions seem to be strongly supported by the literature. As delineated on the previous pages of this review, almost all studies conducted in MND/dementia as well as in nondemented MND patients stress the frontal-executive dysfunction and deficits in social cognition as the most prominent changes in both groups, linking them clearly to the frontal variant FTD. However, the very universality of this consensus might be a source of a systematic bias. In a recent review,[91] identified 12 studies devoted to fluency, 11 to executive functioning, but only 7 to language and 4 to visuoperceptual functions. Moreover, while the executive functions were assessed by a variety of different tests, language testing in most cases was confined to object naming which, as discussed in the language section of this review, is the best-preserved language function in this group.

Looking beyond the present consensus, the notion of a unitary MND/FTD is confronted with several challenges. There seems to be a substantial discontinuity between FTD/dementia and the cognitive changes in nondemented MND patients. FTD/dementia belongs to the most severe and most rapidly progressive dementing syndromes, at times raising the differential diagnosis of Creutzfeldt–Jakob disease. In contrast, the majority of nondemented MND patients have little overt symptoms in their everyday life and very sophisticated tests are needed to uncover subtle deficits. Obviously, this could be due to the fact that both groups have usually been examined separately and further research might demonstrate a more continuous distribution of symptoms across both groups.

More importantly, however, there might exist a qualitative difference between MND/dementia and the classical FTD. First, the frequency of psychotic symptoms mentioned in the earlier descriptions and confirmed in recent studies seems to be higher than we would expect in FTD.[92] Second, although language impairment has not been traditionally regarded as an important feature of MND, studies that examined language functions in a systematic way, found substantial deficits, particularly in language comprehension, often amounting to a full-blown aphasia. As suggested by a recent study,[93] the degree of comprehension deficit in MND/dementia patients is not only higher than in frontal variant FTD, but also in the two aphasic subtypes of FTD: nonfluent progressive aphasia and semantic dementia.

An alternative view of the MND/FTD overlap is that of a “syndromic association,”[50] a co-occurrence of two separate conditions. The fact that they occur together more frequently than we would expect by chance could be explained through shared pathological mechanisms, genetic or environmental risk factors or an interaction of pathologies making one disease a risk factor for the other. However, in the context of MND/FTD this seems to be the least satisfying explanation. On one hand, it does not offer a unifying hypothesis, as does the MND/FTD continuum. On the other hand, it does not account for possible differences between the typical phenotype of MND/FTD and the typical phenotypes of both diseases when they occur in isolation.

The most cautious position, although seemingly going against Ockham’s powerful dictum that “entities should not be multiplied without necessity”, would be to divide the spectrum of MND and FTD into more than two separate entities. Such an approach would distinguish between the classical FTD, the classical MND (which might well include a subtle cognitive dysfunction), and MND/dementia as an independent category. The latter would be characterized by a typical time course (first behavioral, then cognitive, and then motor changes) as well as by the prominence of psychotic and aphasic symptoms. Indeed, in some cases it would be justified to speak of an “MND/dementia/aphasia syndrome.”

Although such a “splitting” rather than “lumping” approach might at first look counter-intuitive, there are signs that this might indeed be the direction that will be taken by future research. After the long battle for the recognition of mental symptoms as an integral part of MND has been won and a robust consensus about an MND/FTD overlap has been reached, the attention of researchers is turning more and more toward the variability and individual differences within the MND/FTD spectrum. Murphy *et al*.[36] propose to divide the nondemented MND patients into two broad categories (albeit with some degree of overlap): the cognitively impaired (ci) and behaviorally impaired (bi). Such an approach has been adopted by the consensus meeting to establish criteria for MND/dementia.[94] With the growing recognition of linguistic dysfunction in MND, it is possible that another category, “language impaired” will be added soon. The gradual shift of emphasis from studies treating MND patients as a homogeneous group to those exploring individual differences in performance is likely to lead to the discovery of distinct subtypes of cognitive and behavioral impairment. Such subtypes would also offer a much better basis for the study of the relation between the cognitive phenotypes and the genotype; a study likely to gain importance with the breathtaking speed of genetic and molecular discoveries in recent years.
